# An Investigation of Colistin Heteroresistance in *Klebsiella pneumoniae* Isolates in Iran

**DOI:** 10.1155/bmri/4239177

**Published:** 2026-02-04

**Authors:** Zohreh Riahi Rad, Zahra Riahi Rad, Hossein Goudarzi, Mehdi Goudarzi, Mohsen Javidi, Javad Yasbolaghi Sharahi, Masoud Kargar, Ali Hashemi

**Affiliations:** ^1^ Department of Microbiology, School of Medicine, Shahid Beheshti University of Medical Sciences, Tehran, Iran, sbmu.ac.ir; ^2^ Thalassemia and Hemoglobinopathy Research Center, Health Research Institute, Ahvaz Jundishapur University of Medical Sciences, Ahvaz, Iran, ajums.ac.ir

**Keywords:** antibiotic resistance, carbapenem-resistant *K. pneumoniae* (CRKP), colistin, heteroresistance, *Klebsiella pneumoniae*, population analysis profiles

## Abstract

**Background:**

According to the World Health Organization 2024 bacterial priority pathogens list, carbapenem‐resistant *Enterobacterales* (CRE) were listed among the critical priority pathogens. Heteroresistance (i.e., a bacterial isolate that appears susceptible but harbors resistant subpopulations) represents a challenge in traditional laboratory testing, which may lead to treatment failure with colistin. This phenomenon has been studied in many bacteria, including *K. pneumoniae*. To our knowledge, this is the first report of colistin heteroresistance in *K. pneumoniae* isolates using population analysis profiles (PAPs) in Iran.

**Methods:**

Between 2019 and 2020, 100 *K. pneumoniae* isolates were collected from various samples of hospitalized patients in Iran. This study primarily determined antibiotic resistance by antimicrobial susceptibility testing. Thereafter, the prevalence of colistin heteroresistance in *K. pneumoniae* isolates was evaluated by the PAP test. Heteroresistant isolates were typed by multilocus sequence typing (MLST).

**Results:**

The MIC test showed that 79 (79%) of the 100 *K. pneumoniae* isolates were susceptible to colistin. Overall, 2/79 colistin‐susceptible isolates were classified as heteroresistant isolates by the PAP method, with a colistin MIC of 0.5 *μ*g/ml. Importantly, after 5 serial passaging on colistin‐free plates, there was no increase in the MIC of the colistin‐resistant subpopulations, showing that heteroresistance cases were unstable. MLST revealed that heteroresistant isolates belong to ST377 and ST15.

**Conclusions:**

In conclusion, the current study contributes to our understanding of the challenges posed by heteroresistant isolates in clinical laboratories. Since heteroresistant isolates may be misidentified as susceptible by standard tests, these findings raise concerns regarding the interpretation of colistin susceptibility results.

## 1. Introduction

Bacterial antimicrobial resistance (AMR) is increasingly recognized as a 21st‐century global health concern [1]. If the advancement of AMR is not restrained, it could make many bacterial pathogens considerably more lethal [2]. An investigation conducted by the UK Government has shown that by 2050, AMR could cause 10 million deaths annually [1]. A study published in *The Lancet* demonstrated that in 2019, approximately 1.27 million deaths were due to AMR; this rate of death is higher than the number of deaths caused by HIV/AIDS (864,000 deaths) and malaria (643,000 deaths) [2]. Based on the latest World Health Organization (WHO) update of the bacterial priority pathogens list in 2024, carbapenem‐resistant *Enterobacterales* were located in a critical priority group [3].

*Klebsiella pneumoniae* is an opportunistic gram‐negative bacterial pathogen belonging to the *Enterobacterales* order and is a serious nosocomial pathogen. Carbapenems have been used for infections due to *K. pneumoniae* producing extended‐spectrum *β*‐lactamase (ESBL) [4]. In recent years, an increasing prevalence of carbapenem‐resistant *K. pneumoniae* (CRKP) has been reported globally [5].

The inaccessibility of new antibacterial agents to tackle CRKP infections has revived the utilization of polymyxin B and colistin (CO) [6]. CO targets the lipid A moiety of lipopolysaccharide (LPS) in gram‐negative pathogens with bactericidal activity and disrupts the bacterial cell membrane. Many investigations have found that CO resistance in *K. pneumoniae* is due to mutations in the two‐component systems PmrAB and PhoPQ, alterations to the negative regulator MgrB, and the mobile CO resistance gene (mcr‐1), which encodes a phosphoethanolamine transferase that modifies lipid A [5, 7].

Identical bacterial cells in a population can display phenotypic heterogeneity regarding antibiotic susceptibility, which results in difficulties in unequivocally classifying bacteria as susceptible or resistant [8]. While bacterial resistance is typically a stable characteristic (e.g., horizontal gene transfer or mutation in native genes), this feature can also be unstable. This instability occurs only in a small fraction of the population, leading to phenotypic heterogeneity within the bacterial population [9].

An instance of this phenomenon is antibiotic heteroresistance, and one of the major concerns is the evolution of distinct resistance in bacterial isolates. The most comprehensive definition of heteroresistance is the presence of a heterogeneous population of bacteria with one or several subpopulations exhibiting higher levels of antibiotic resistance than the main population. This phenomenon is identified in the majority of antibiotic classes, including carbapenems and the last‐resort polymyxins (polymyxin B and CO). Many studies have investigated heteroresistance to polymyxin in gram‐negative bacteria, including *Salmonella enterica* subsp. *enterica* serovar Typhimurium, *Klebsiella* spp., *Pseudomonas aeruginosa*, *Acinetobacter baumannii,* and *Enterobacter* spp. [8]. Alarmingly, the prevalence of CO‐heteroresistant *K. pneumoniae* has recently increased.

Antibiotic susceptibility tests (AST) such as the disk diffusion, MIC, and Etest are typically unable to identify heteroresistant strains due to the phenotypic and genetic instability of this phenomenon, and might be misclassified as susceptible since a small fraction of the resistant subpopulation is overlooked [10, 11]. The population analysis profile (PAP) test is the most reliable and quantitative method to detect the resistant subpopulation [8].

Our knowledge of the impact of heteroresistance on patient treatment is incomplete, and additional research is required to address this large gap. Recent experimental and clinical findings indicate that multidrug‐resistant (MDR), extensively drug‐resistant (XDR), or pandrug‐resistant (PDR) strains are not the only reasons for treatment failure. The phenomenon of heteroresistance can be the cause of poor treatment results, as strains are misclassified as susceptible, which frequently leads to inappropriate use of antibiotics [10]. The mechanism of heteroresistance to CO in bacteria is different, including the activation of two‐component systems PmrAB and PhoPQ in bacteria *K. pneumoniae*, *A. baumannii*, *P. aeruginosa*, and *Enterobacter cloacae*, biofilm formation and capsule hyperproduction in *K. pneumoniae*, soxRS‐regulated overexpression of the acrAB‐tolC efflux pump in *Enterobacter asburiae* and *E. cloacae* [12]. We aimed to investigate the existence of CO‐heteroresistance among *K. pneumoniae* isolates in Iran, and highlight the importance of improving new procedures of heteroresistance identification.

## 2. Materials and Methods

### 2.1. Bacterial Strains

In total, 100 nonduplicate *K. pneumoniae* isolates were collected for this study between 2019 and 2020. These isolates were gathered from various clinical samples of hospitalized patients in six cities of Iran: Tehran, Ahvaz, Bandar Abbas, Ilam, Tabriz, and Qom. The identification of *K. pneumoniae* clinical isolates was carried out using standard biochemical methods, including growth on the Simmons′ citrate agar, indole production, urease production on urea agar, motility on the SIM medium, reaction in the MR‐VP tests, reaction on the TSI culture medium, and the oxidase test [13]. The *K. pneumoniae* isolates were stored in trypticase soy broth (TSB) with 20% glycerol at −70°C before the analysis described in this study. All media were purchased from Merck, Darmstadt, Germany.

### 2.2. Antimicrobial Susceptibility Testing

We carried out standard AST using the Kirby–Bauer disk diffusion method on Mueller–Hinton agar plates (Merck, Germany), consistent with the 2023 Clinical and Laboratory Standards Institute (CLSI) breakpoints [14]. Several common antibiotic disks (Mast, Company) were used, including ampicillin (AP, 10 *μ*g), piperacillin‐tazobactam (PTZ, 110 *μ*g), imipenem (IMI, 10 *μ*g), meropenem (MEM, 10 *μ*g), doripenem (DOR, 10 *μ*g), ertapenem (ETP, 10 *μ*g), ceftazidime (CAZ, 30 *μ*g), cefotaxime (CTX, 30 *μ*g), cefepime (CPM, 30 *μ*g), gentamicin (GEN, 10 *μ*g), amikacin (AK, 30 *μ*g), ciprofloxacin (CIP, 5 *μ*g), levofloxacin (LEV, 5 *μ*g), tetracycline (T, 30 *μ*g), minocycline (MN, 30 *μ*g), aztreonam (ATM, 10 *μ*g), trimethoprim‐sulfamethoxazole (TS, 25 *μ*g), and fosfomycin (FOS, 200 *μ*g). Quality control for AST was conducted using the *E. coli* ATCC 25922 strain [15]. In compliance with the 2023 CLSI guidelines, minimum inhibitory concentrations (MICs) for the antimicrobial agents of IMI, MEM, CAZ, CTX, CIP, and CO (Sigma‐Aldrich) were determined using the broth microdilution method [14]. Since CLSI does not define susceptible breakpoints for CO, the CO‐MIC values were interpreted according to the epidemiological cut‐off (ECOFF) values provided by the instructions of the European Committee on Conducting Antimicrobial Susceptibility Testing (EUCAST, 2023) (http://www.eucast.org/clinical_breakpoints).

### 2.3. Identification of Carbapenemase and Metallo‐*β*‐Lactamase (MBL)

The Modified Carbapenem Inactivation Method (mCIM) and the EDTA‐enhanced Carbapenem Inactivation Method (eCIM) were applied to detect carbapenemase and differentiate MBLs from serine carbapenemase in heteroresistant isolates, respectively, in agreement with the 2023 CLSI guidelines [14].

### 2.4. Identifying ESBL‐Producing Heteroresistant Isolates

The combination disk diffusion test (CDDT) was performed to assess ESBLs in the resistant subpopulations. Thus, CTX (30 *μ*g) and CAZ (30 *μ*g) with CTX + clavulanic acid and CAZ + clavulanic acid (10 *μ*g) per disk were employed. All disks were purchased from Mast Group, Merseyside, United Kingdom [16]. *K. pneumoniae* ATCC 700603 and *E. coli* ATCC 25922 were used as positive and negative controls, respectively.

### 2.5. CO‐Heteroresistance Assay

**Figure 1:**
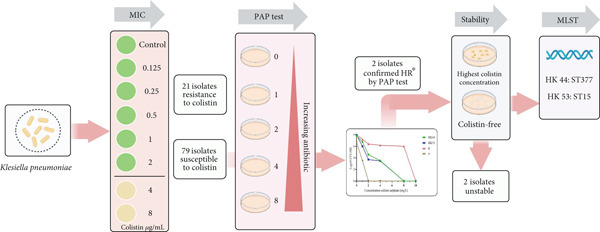
Workflow for detecting heteroresistance *K. pneumoniae* isolates.  ^∗^HR: Heteroresistance.

In this study, the PAP assay was utilized as the reference method to detect CO heteroresistance among *K. pneumoniae* isolates [9, 17]. One hundred microliters (100 *μ*L) of dilutions of the bacterial cell suspension were plated on Mueller–Hinton agar (MH agar) plates with or without increasing concentrations of CO: 0.5, 1, 2, 4, 8, 16, and 32 mg/L. Plates were then incubated at 37*°*C and CFU were enumerated after 48 h. Considering the EUCAST breakpoint of colistin MIC values for *K. pneumoniae*, CO heteroresistance can be characterized as follows: (a) a CO‐susceptible isolate (MIC of ≤ 2 *μ*g/ml), with subpopulations growing in the presence of > 2 *μ*g/mL CO [17]; (b) the growth of a CO‐resistant subpopulation of cells at a CO concentration that is at least eight times higher than the MIC of the main population, with a frequency of 1 × 10^−7^ [8] (Figure 1).

The frequency of CO heteroresistance was measured using the following formula [18]:

Frequency=CFU/mL colistin plateCFU/mL colistin−free plate

Subsequently, the stability of the resistant subpopulations by selecting a single colony from the plate with the highest antibiotic concentration was evaluated by five serial passaging on CO‐free plates. Then, the CO‐MIC was reassessed.

### 2.6. Multilocus Sequence Typing (MLST)

Sequence types (STs) of the CO‐resistant subpopulations were determined using housekeeping genes: *tonB*, *rpoB*, *infB*, *phoE*, *pgi*, *mdh*, and *gapA* in MLST [19].

## 3. Results

### 3.1. Bacterial Isolates

**Figure 2:**
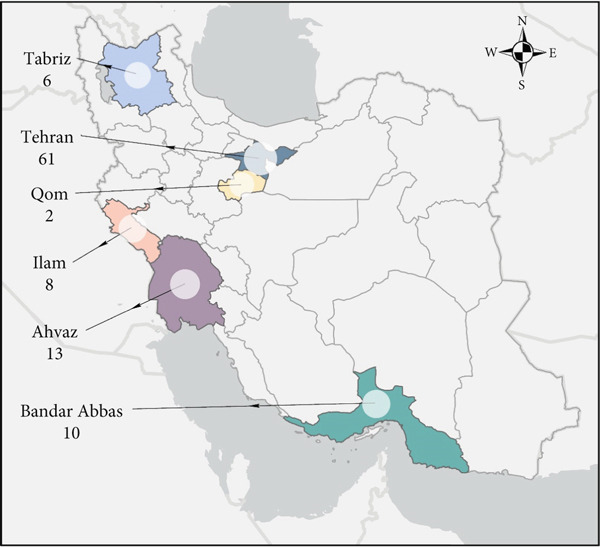
A total of 100 *K. pneumoniae* isolates collected from Iran.  ^∗^The map was created using ArcGIS Pro.

The clinical *K. pneumoniae* isolates (*n* = 100) were collected between 2019 and 2020 from different cities of Iran, including Tehran (*n* = 61), Ahvaz (*n* = 13), Bandar Abbas (*n* = 10), Ilam (*n* = 8), Tabriz (*n* = 6), and Qom (*n* = 2). Figure 2 Of the 100 isolates, 60 (60%) were isolated from men, and 40 (40%) from women. Furthermore, 14 (14%) of the total isolates were obtained from children. The majority of isolates originated from sputum 34(34, 34%) and urine (32, 32%), followed by blood (20, 20%), wound (7, 7%), ascitic fluid (2, 2%), throat culture (2, 2%), trachea (1, 1%), bronchoalveolar lavage (1, 1%), and drainage fluid (1, 1%). Most isolates were obtained from the intensive care units (ICU) (53, 53%), burn (15, 15%), and surgery (12, 12%) wards, followed by internal (8, 8%), oncology (5, 5%), cardiology (4, 4%), and dialysis (3, 3%).

### 3.2. Antimicrobial Susceptibility

From the data in Table 1, it is apparent that AP 99% (99), CTX 95% (95), and PTZ 80% (80) have a high resistance rate among *K. pneumoniae* isolates. Furthermore, this table reveals that MN 30% (30), FOS 42% (42), and T 45% (45) had the lowest resistance rate. Overall, the AST results indicated that 70 isolates showed resistance to *β*‐lactam antibiotics. Conforming to the EUCAST 2023 guidelines, of the total isolates that were evaluated by the MIC test, 21 (21%) were classified as resistant to CO with the MIC of > 2 *μ*g/mL, and 79 (79%) of isolates were susceptible to CO with the MIC of ≤ 2 *μ*g/mL (note: the resistant isolates were not further analyzed). Table 1 Besides, the highest number of CO‐resistant isolates (12, 12%) was observed in Tehran.

**Table 1:** Profiles of antibiotic resistance of the 100 *K. pneumoniae* isolates.

**Antibiotics**	**Disk diffusion (** **n** = *%* **)**			**MIC (** **n** = *%* **)**		
	**S**	**I**	**R**	**S**	**I**	**R**
Penicillins						
Ampicillin	—	1	99	—	—	—
*β*‐lactam combination						
Piperacillin‐tazobactam	11	9	80	—	—	—
Carbapenems						
Meropenem	21	11	68	18	10	72
Imipenem	30	5	68	22	10	68
Doripenem	9	22	69	—	—	—
Ertapenem	28	3	69	—	—	—
Cephalosporins						
Ceftazidime	16	14	70	27	2	71
Cefotaxime	—	5	95	5	11	84
Cefepime	18	3	79	—	—	—
Aminoglycosides						
Amikacin	22	27	61	—	—	—
Gentamicin	21	19	60	—	—	—
Fluoroquinolones						
Ciprofloxacin	6	8	86	13	5	82
Levofloxacin	17	17	66	—	—	—
Tetracyclines						
Tetracycline	53	2	45	—	—	—
Minocycline	48	22	30	—	—	—
Monobactams						
Aztreonam	22	12	66	—	—	—
Folate pathway antagonist						
Trimethoprim‐sulfamethoxazole	12	2	86	—	—	—
Fosfomycins						
Fosfomycin	48	10	42	—	—	—
Lipopeptides						
Colistin	—	—	—	79	—	21

Abbreviations: I, intermediate; R, resistant; and S, susceptible.

### 3.3. CO‐Heteroresistance and MLST Analysis Results

**Figure 3:**
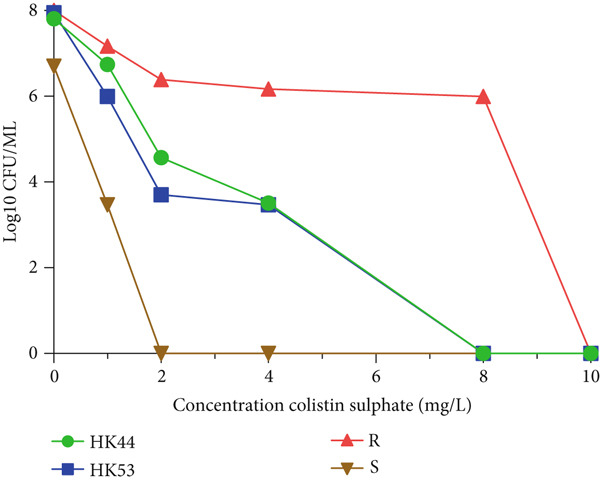
Population analysis profiles of two *K. pneumoniae.*

Based on the PAP test result, we confirmed that of 79 CO‐susceptible *K. pneumoniae* isolates, two minor CO‐resistant subpopulations (HK44 and HK53) were identified. Noticeably, the initial MIC result for both parental isolates was 0.5 mg/L. These two CO‐resistant subpopulations grew in the presence of CO at concentrations of 4 mg/L. Figure 3. The findings revealed that the growth of HK44 and HK53 isolates had an eightfold increase in CO‐MICs than their respective parental isolates. The frequencies of heteroresistant subpopulations in HK44 and HK53 were 5 × 10^−5^ and 3.3 × 10^−5^, respectively. Notably, after serial subculturing (five daily) on CO‐free MH plates, there was no increase in the MIC of the CO‐resistant subpopulations. This suggests the instability of the heteroresistant phenotype. Table 2 The HK44 and HK53 subpopulations belonged to different STs, ST‐377 and ST‐15, respectively (Table 2, Figure 1). Both heteroresistant isolates were ESBL producers. According to the mCIM test and the eCIM test, HK44 and HK53 produced carbapenemase and serine *β*‐lactamase.

**Table 2:** Data of the two heteroresistance isolates.

**Strain**	**Gender**	**City**	**Ward**	**Culture source**	**Broth MIC (*μ*g/ml)**	**Highest concentration of growth in PAPs (mg/L)**	**Frequency**	**MIC for resistant colonies before daily passages**	**MIC for resistant colonies after 5 days passages**	**ST**	**Stability**
HK44	Woman	Tehran	ICU	Throat culture	0.5	4	5 × 10^−5^	16	1	377	Unstable
HK53	Man	Tehran	Burn	Urine	0.5	4	3.3 × 10^−5^	8	0.5	15	Unstable

It was interesting to note that HK44 and HK53 were resistant to many classes of both *β*‐lactam and non‐*β*‐lactam antibiotics. Both HK44 and HK53 isolates had resistance to all carbapenem groups of antibiotics including MEM, IMI, ETP, and DOR (Table 3).

**Table 3:** Profiles of antibiotic resistance of the two heteroresistance isolates.

**Strains**	**Disk diffusion**																		**MCIM**	**ECIM**	**ESBL**
	**IMI**	**MEM**	**DOR**	**ETP**	**GM**	**LEV**	**CTX**	**CAZ**	**CPM**	**MN**	**T**	**ATM**	**AK**	**PTZ**	**TS**	**CIP**	**AP**	**FO**			
HK44	*R*	*R*	*R*	*R*	*R*	*R*	*R*	*R*	*R*	*I*	*S*	*R*	*R*	*R*	*R*	*R*	*R*	*I*	**+**	Serine	**+**
HK53	*R*	*R*	*R*	*R*	*R*	*R*	*R*	*R*	*R*	*S*	*S*	*R*	*R*	*R*	*R*	*R*	*R*	*S*	**+**	Serine	**+**

## 4. Discussion

The WHO has warned of the spread of AMR, calling for a globally coordinated strategy to address this threat. If untackled, the spread of AMR could be one of the major lethal factors in the future [2, 20]. Because of limited treatment options, the emergence of CO resistance has made it a formidable challenge to tackle CRKP [21].

Adding to these concerns, the emergence of CO heteroresistance has posed a serious threat to the clinical use of CO. It should be noted that the first description of heteroresistance by Alexander HE et al. was described in the 1940s [22]. Heteroresistance is a phenotype that displays population‐wide variable susceptibilities to a specific antibiotic [8, 23], which has also been studied in *K. pneumoniae* isolates [5, 24]. The issue has grown in importance in recent investigations, and this study provides important insights into the understanding and detection of heteroresistance.

The ASTs evaluate the growth of the entire bacterial community exposed to different antibiotics to categorize whether the isolate is resistant or susceptible, but cannot distinguish heteroresistant bacteria [25]. Since accurately identifying the heteroresistance by reference methods such as gradient strip, disk diffusion, and broth microdilution is difficult, it might have an impact on the treatment of clinical infection [26].

The PAP enables the visualization and appearance of heteroresistant populations [27]. In line with the PAP results, this study identified two CO‐heteroresistant strains (HK44 and HK53) among the 79 CO‐susceptible *K. pneumoniae* isolates using a microdilution broth test, with MIC values of 0.5 *μ*g/mL. Our findings align with previous studies and suggest that heteroresistant isolates may be misclassified as susceptible by the MIC test [17, 28–30], which is likely due to the low frequency of resistant subpopulations that cannot be detected [26]. Furthermore, Ozturk et al. demonstrated that the discrepancy in AST results might be attributed to the heteroresistance phenomenon [31].

In general, few epidemiological data about the prevalence of heteroresistance are available, and Huang stated that: “the prevalence of heteroresistance is underreported and it is underappreciated” [6, 10, 32]. In this regard, our study provides more information on heteroresistance that would help researchers in this matter. To our knowledge, this is the first description of CO heteroresistance in *K. pneumoniae* isolates in Iran. In this study, out of the 79 CO‐susceptible *K. pneumoniae* isolates tested, 2.5% (2/79) were identified as heteroresistant to CO. Cheong et al. also reported a low percentage (1.3%) of the CO‐heteroresistant phenotype, and among the 231 CO‐susceptible *K. pneumoniae* isolates, three isolates were heteroresistant to CO [5]. However, Poudyal et al. reported that among the 21 K*. pneumoniae* isolates tested, 71.4% (15/21) were heteroresistant to CO [29]. Also, Meletis et al. showed that 12 of the 20 *K. pneumoniae* isolates exhibited heteroresistance [33]. Band et al. and Weng et al., respectively, have found 8.4% (24/286) and 6.2% (28/455) of CO‐susceptible *K. pneumoniae* isolates that show heteroresistance to CO [34, 35]. Recently, Braspenning et al. have shown that in 288 CO‐susceptible MDR *K. pneumoniae* isolates that were randomly selected, 37.5% (*n* = 108) were CO heteroresistant [18]. A possible explanation for this contradiction in results may be due to the complexity of detecting heteroresistance and the different detection methods applied.

The CO‐MIC of two heteroresistant isolates decreased after five serial passages on CO‐free plates and reverted to the susceptible phenotype after the drop of antibiotic pressure, which suggests the importance of stability measurement because this instability leads to difficulties in our ability to detect heteroresistance [6, 10, 26].

One major quandary in heteroresistance concerns its effective role in treatment failure, which has been investigated in some animal studies [6]. A study conducted by Band et al. in the United States in 2017 showed that CO was effective in saving mice infected with an *E. cloacae* susceptible strain, whereas mice infected with the heteroresistant isolates failed CO treatment and did not survive [36]. The retrospective study by Moosavian et al. revealed that carbapenem heteroresistance in *A. baumannii* caused treatment failure to CO in a patient with meningitis. Notably, it was the first report of CO‐heteroresistance in Iran [37]. The study by Band et al. indicated CO has not been an effective treatment for *K. pneumoniae* heteroresistance‐infected mice and were unable to survive [28]. Other important findings were that antibiotic combinations might have an impact on infections related to heteroresistant *K. pneumoniae* [10, 38]. Because of limited access to advanced molecular equipment and the restricted duration of the study, genetic characterization of the heteroresistant isolates was not feasible.

## 5. Concluding Remarks

This study reveals that identifying CO‐heteroresistant phenotype represents a threatening challenge for treating infections caused by CRKP and has likely been underappreciated. Considering that this phenomenon may not be detected by established procedures (disk diffusion and broth microdilution) in clinical microbiology laboratories, the present study highlights the need for methods to assess its clinical and standard operational definition. Although the PAP test is reliable, it is time‐consuming and complex; therefore, new diagnostic tests that are sensitive enough and have high reproducibility to diagnose heteroresistance are needed. A notable limitation of this research is that the genetic alterations in the heteroresistant strains were not assessed. Therefore, further research on this topic needs to be undertaken so that the association between heteroresistance and clinical outcomes is more clearly understood. Increased CO resistance leads to the limitation of available treatment options for managing highly resistant infections. We suggest that CO be used cautiously as a last resort option to control CRKP infections.

## Ethics Statement

Before undertaking the investigation, the Ethics Board of Shahid Beheshti University of Medical Sciences approved this study with the reference number IR.SBMU.MSP.REC.1397.629. To ensure participant privacy, no personal data were gathered or included in the research, and all participants remained anonymous.

## Disclosure

All authors read and approved the final manuscript.

## Conflicts of Interest

The authors declare no conflicts of interest.

## Author Contributions

Zoh.R.R., Zah.R.R., M.J., and A.H. conceived, designed, and performed the experiments, and analyzed the data. M.K. designed the figures. Zoh.R.R. and Zah.R.R. wrote the paper. H.G., M.G., and J.Y. contributed to the interpretation of results, provided critical revisions to the manuscript, and helped refine the final discussion. Additionally, J.Y. contributed to data collection.

## Funding

This study was supported by the School of Medicine, Shahid Beheshti University of Medical Sciences (10.13039/501100022299, 17674).

## Data Availability

The data that support the findings of this study are available from the corresponding author upon reasonable request.
